# 

**Published:** 1982

**Authors:** 


JULY/OCTOBER 1982

VOLUME 97 iii/iv

NUMBER ~~359/360~~ 363/364

Published Quarterly

Annual Subscription £8 Post Free

### BRISTOL MEDICO-CHIRURGICAL JOURNAL

Contents

3 Editorial

Cossham Medical Society

Bristol Medic-Legal Society

41 Examination Results

Established 1883

### BRISTOL MEDICO-CHIRURGICAL JOURNAL

Incorporating the Medical Journal of the South West

### Editorial Committee

EDITOR
*Mr. M. G. Wilson*
*Litfield House, Clifton, Bristol, BS8 3LS*
ASSISTANT EDITOR
*Dr. G. R. Tribe*
*Southmead Hospital, Bristol, BS10 5NB*
MEMBERS
*Dr. M. B. Lennard*
*Dr. D. W. Wright*
*The Hon. Treasurer of the Society*
*The Hon. Secretary of the Society*
SECRETARY
*Mr. B. P. Jones, The Library, Medical School, University Walk, Bristol, BS8 1TD*

### Bristol Medico-Chirurgical Society

PRESIDENT 1980/81
*Dr. N. J. Brown*
PRESIDENT ELECT
*Dr. R. P. Warin*
HONORARY SECRETARY
*Dr. H. M. Norman*
*Hillside, Vicarage Lane, Barrow Guerney*
HONORARY TREASURER
*Dr. J. Penry*
*Southmead Hospital, Bristol, BS10 5NB*

The Society was founded in 1874. In 1883 publication of the Journal began and has continued quarterly ever since then. During the years 1949 to 1962 it appeared under the title of 'The Medical Journal of the South West'.

### Notice to Contributors

*The Journal is especially concerned to provide a place for the recording of medical thought, interests, and practice in and around Bristol. It therefore welcomes articles that, as well as being of immediate interest to members, will document the local and contemporary medical scene for our successors.*

*Original articles are invited provided that they have not been published elsewhere. Illustrations should be supplied ready for reproduction. Line drawings should be in Indian ink on firm white paper or board. The author will be informed if it is found necessary to ask him to pay for the printing of photographs, etc.*

*The following contributions will be especially welcome: notes of forthcoming events, meetings held, degrees conferred, staff appointments, honours and public appointments and other local news.*

*Advice on preparation can be obtained from the Editor.*

Printed by the University of Bristol Printing Unit

### Programmes of Societies, 1982-83

### COSSHAM MEDICAL SOCIETY

Programme
1982–1983

President: Dr. Chris Burns-Cox

Meetings are held at 8.30 p.m. at Frenchay Postgraduate Centre

1982

Tuesday 5th October
Presidential address.
Dr. Chris Burns-Cox.

Tuesday 2nd November
"Edward Jenner."
Canon Gethin Jones.

Saturday, 27th November
Annual Cocktail Party, 8.00 p.m.
Frenchay Postgraduate Centre.

Tuesday 7th December
"Childbirth – where and why."
Professor Gordon Stirratt.

1983

Tuesday 11th January
"Oxfam and disaster medicine."
Dr. Tim Lusty.
Oxfam Disaster Relief.

Tuesday 1st February
"Recollections of a magpie."
Dr. Max Valentine.

Tuesday 1st March
"The work of the Anti-Slavery Society today."
Mr. Peter Davies.
Secretary of The Anti-Slavery Society.

Tuesday 5th April
A.G.M. and a Speaker.

**ALL MEETINGS OPEN MEETINGS**

**Honorary Secretaries:**
Dr. Michael J. Doyle, 267 Soundwell Road, Bristol Tel. 673630
Dr. Bernard G. Whiteside, 130 High Street, Hanham, Bristol. Tel. 675201

**Honorary Treasurer:**
Dr. J. Richard Bernard, Fishponds Health Centre, Bristol. Tel. 656281

### BRISTOL MEDICO LEGAL SOCIETY

The meetings will be held in the School of Nursing at the Bristol Royal Infirmary and a buffet supper will be available from 6.30 p.m.

1982

Thursday 16th September. 8.00 p.m.
**What you eat is my Business**
Mr. Braxton Reynolds,
B.Sc., M.Chem.A., M.R.S.C.
Public Analyst, Devon County Council

Thursday 18th November. 8.00 p.m.
**The Development of Victims Support Schemes**
Miss Helen Reeves, *National Officer*
National Association of Victims Support Schemes

1983

Thursday 20th January. 8.00 p.m.
**Medicine and the Law**
Dr. John Wall, *Assistant Secretary*
Medical Defence Union

FRIDAY 25th FEBRUARY
The Royal West of England Galleries
**ANNUAL DINNER**
Professor Alan Usher, O.B.E., F.R.C.Path.
*Head of Department of Forensic Pathology Sheffield University, Consultant Pathologist to the Home Office*

Thursday 17th March. 8.00 p.m.
**The Right to Life**
Mr. Michael Bell, M.A.

Thursday 19th May. 8.00 p.m.
**Members' Papers**

Friday 2nd July
**Summer Social Gathering.**

### University of Bristol Faculty of Medicine

**THE DEGREE OF DOCTOR OF MEDICINE**
**June 1982**
**Dissertations Approved**

David HONEYBOURNE
David Gordon Islay SCOTT
John Richard THORNTON
Yat-Cheung WONG

**THE DEGREE OF MASTER OF SCIENCE IN MEDICAL SCIENCES**
**June 1982**
**Dissertations Approved**

Jeanette Rose TODD
Elizabeth Jane YOUNG

**THE DEGREE OF DOCTOR OF PHILOSOPHY**
**June 1982**
**Dissertations Approved**

Timothy John GRUFFYDD-JONES
Nikolaos KOTSANOS

**FINAL EXAMINATION FOR THE DEGREES OF M.B., Ch.B.**
**June 1982**

**WITH HONOURS**
Ian Colin John Walker BOWLER
with Distinction in Mental Health
Helen Mary Kirkman BROWN
with Distinction in Child Health
Susanna Ruth HILL
Erica Mary JONES
with Distinction in Mental Health
Peter Ian MANSELL
with Distinction in Pathology
Simon Richard PAGE
Elizabeth Ann SEPHTON
with Distinctions in Medicine and in Obstetrics and Gynaecology
Paul John STRICKLAND
with Distinction in Mental Health
Philippa SWAYNE
with Distinction in Obstetrics and Gynaecology

**PASS**
Elizabeth Francis Maitland ADAMS
James Alan Olof AHLQUIST
Phillip Martyn ALDERMAN
Antony Mark ALFORD
Stephen John ALVIS
Shiv ANAND
Edmund George ATKINSON
Eric John BATER
Helen Jane BAYLISS
Sara Catherine BEATTIE
Claire Elizabeth BENSON
Carolyn Mary BURGESS
David Michael BYFIELD
with Distinction in Obstetrics and Gynaecology
Andrew Jonathan CARR
Peter Martin CARTER
Antoinette Hazel CHALLEN
Vincent Louis CHAMARY
Tsungayi CHIPATO
Matthew Guy Parry CLARKE
Nigel Morrison CLAYTON
Ann Patricia COCKBURN
Gillian COLLINS
Jane Elizabeth COWKING
Amanda Louise COX
Bernita Ann CROSS
Peter Michael CUMBER
Edward Phillip Paul CURTIS
Christine Elizabeth DAVIES
Gillian Carolyn DEGENS
Mervyn Terence DENT
Clare Nicola DOUGLAS
Clare Stephanie DUNKLEY
Mark Peter DYKE
Jonathan Michael EDWARDS
Carolyn ELLIS
Sean Anthony Greensmith ELYAN
Robert James ETHERINGTON
Elizabeth Rothwell GERMANY
Malcolm Seyton HAMILTON
with Distinction in Child Health
William Trevor HAMILTON
Sucha HAMPAL
Julian Martin HANCOCK
Robert John Alexander HARTE
John HERBETKO
Josephine HERDMAN
Therese Mary HESKETH
Patricia Ann JACOB
Pauline Anne JOHNSON
David Newton JONES
Kathryn Anne JONES

Malcolm Rosser JONES
Anil Kumar JOSHI
Barbara Anne Katherine KENNEY
James Hugh LARCOMBE
Elizabeth LARKIN
Graham LENG
Chon Kar LEOW
Sian Elspeth LEWIS
Lucy Caroline MACAULAY
Gillian Anne MACLEOD
Philip Gerard McCARTHY
Mark Robert McCARTNEY
Fiona Kathleen McVEY
Jennifer Mary MARSON
Martin Stuart MILLS
Michael William Musgrave MORGAN
Helen Jane MUTCH
Christopher James NAGLE
Nazlin Hussein NANJI
David Nicholas NEWELL
Hyacinth Kuma NKUO
Alison Frances NORMAN
John Hughes OWEN
Stuart Andrew PARKER
David Ralph PARTRIDGE
Sally Elizabeth PELLER
Michael Henry James PIMM
Judith Mary PROBERT
Kumari Nalini RAMFUL
Clare Diana REDSTONE
Nicholas Paul RING
David Michael ROBERTS
Alexandra Jane ROBINSON
Caroline Jane ROSE
Alison Pamela ROUND
Steven Fraser Owen SCOTT
Nigel Henry Leighton SELLARS
Daniel Sean SELSBY
Simon Charles SHEARD
Eileen Patricia SHEPHERD
Daniel Kenneth SMITH
Lindsay Fredrick Paul SMITH
Nicholas Milward SMITH
Christopher John SPELLER
Richard Neil STEPHENSON
Rosemary Jane SYKES
Eryl Ann THOMAS
Christine Mary WARLOW
Jennifer Neale WEBB
David John WEBBORN
Amanda Jane WESTON
David James WILLIAMSON
Emily WILLS
Wai Lup WONG
Catherine Elizabeth WOOD
Patricia Yvonne Ashley WOOD
with Distinction in Community Health
Guy Mark WORSDALL
Paul Anthony YATES

